# 

**Published:** 1818-03

**Authors:** 


### CORRESPONDENCE.

Mr. Cunningham's Paper on a new Mode of Treating large Abscesses, will appear in our next; as will also a Review of the Transactions of the Fellows and Licentiates of the King's and Queen's College of Physicians in Ireland.

The Paper on West India Fever, from the Antelope, is not yet come to hand, but the private letter has been received.

